# Peri‐mortem arrhythmias in the non‐cardiac intensive care unit

**DOI:** 10.1111/nicc.13097

**Published:** 2024-06-03

**Authors:** Iva Okaj, Maria E. Vadakken, Emilie P. Belley‐Côté, Alexandra P. Lengyel, Anand S. Rai, Rubani S. Suri, Jeff S. Healey, William F. McIntyre

**Affiliations:** ^1^ McMaster University Hamilton Ontario Canada; ^2^ University of Manitoba Winnipeg Manitoba Canada; ^3^ McMaster University and Population Health Research Institute Hamilton Ontario Canada; ^4^ Nova University Fort Lauderdale Florida USA

**Keywords:** arrhythmias, atrial fibrillation, death, ECG, organ donation

## Abstract

**Background:**

Cardiovascular failure is recognized as a common final pathway at the end of life but there is a paucity of data describing terminal arrhythmias.

**Aim:**

We aimed to describe arrhythmias recorded peri‐mortem in critically ill patients.

**Study design:**

We enrolled intensive care unit patients admitted to two tertiary Canadian medico‐surgical centres. Participants wore a continuous electrocardiogram (ECG) monitor for 14 days, until discharge, removal or death. We recorded all significant occurrences of arrhythmias in the final hour of life.

**Results:**

Among 39 patients wearing an ECG monitor at the time of death, 22 (56%) developed at least 1 terminal arrhythmia as adjudicated by an arrhythmia physician: 23% (*n* = 9) had ventricular fibrillation/polymorphic ventricular tachycardia, 18% (*n* = 7) had sinoatrial pauses, 15% (*n* = 6) had atrial fibrillation and 13% (*n* = 5) had high‐degree atrioventricular block. Five participants (13%) developed multiple arrythmias.

**Conclusions:**

Arrhythmias are common in dying critically ill patients. There is a roughly even distribution between ventricular arrhythmias, atrial fibrillation, sinus node dysfunction and atrioventricular block.

**Relevance to Clinical Practice:**

The results of this study may be most useful for critically ill patients who are organ donation candidates. The appearance of arrhythmias may serve as a marker of change in clinical status for organ donation teams to plan mobilization efforts. In participants who are sedated or intubated, arrhythmias could be a surrogate marker for respiratory or neurologic changes.

**What is known about the topic**

- Electrophysiological disturbances may occur as part of the common final pathway at the end of life.
- However, the occurrence and characteristics of terminal arrhythmias have not been well‐characterized to date.

**What this paper adds**

- Arrhythmias were found to be common in dying critically ill patients.
- There was a simialr distributuion between ventricular arrhythmias, atrial fibrillation, sinus node dysfunction and atrioventricular block across different etiologies of death.

## 1 INTRODUCTION

Cardiovascular failure is recognized as a common final pathway at the end of life. However, there is a paucity of data describing terminal arrhythmias associated with different modes of death.^1^ Describing frequencies of new‐onset terminal arrhythmias in critically ill patients may offer insights into mechanisms of death. Our objective was to describe arrhythmias in the final hour of life.

## 2 METHODS

We previously reported the design and primary results of the atrial fibrillation occurring transiently with stress (AFOTS) incidence prospective cohort study.^2^, ^3^ In brief, we enrolled intensive care unit (ICU) patients admitted to two tertiary Canadian medico‐surgical centres. Participants were at least 65 years old or between the ages of 50–64, with one or more of the following risk factors: congestive heart failure, hypertension, age ≥ 75, diabetes and stroke/transient ischemic attack.^4^ We excluded patients with a history of atrial fibrillation (AF), those admitted with a primary cardiovascular diagnosis, those not screened within 12 h from admission, those with electrocardiogram (ECG) electrode adhesive allergies, those in whom the 14‐day monitor was expected to interfere with care, those with sleep apnoea admitted to the ICU solely for postoperative monitoring and those with life expectancy or transfer expected within 12–24 h.^2^ Participants wore an ECG patch monitor for 14 days, until discharge, removal or death. We followed participants in hospital and up to 30 days. The local ethics board approved this study.

We defined clinically significant peri‐mortem rhythms as those that occurred for at least 30 s in the final hour of life. We recorded all significant occurrences of the following waveforms: sinoatrial pauses, AF, ventricular fibrillation/polymorphic ventricular tachycardia (VF/PMVT) and atrioventricular (AV) blocks. An arrhythmia physician confirmed all tracings.

## 3 RESULTS

Among 61 participants who died during the study, 39 were wearing the ECG patch at the time of death (Table 1 **)**. Monitoring ranged from 3 h to 11 days and 2 h.

**TABLE 1:** Characteristics of study participants who were wearing an ECG monitor at the time of death.

Characteristic	
Total	39
Age in years (mean [SD])	76.3 (8.5)
Female sex (*n* [%])	13 (33%)
CHA_2_DS_2_‐VaSC score median [Q1–Q3])	3 (3–4)
APACHE II score median [Q1–Q3])	22 (16–25)
BMI (median [Q1–Q3])	26 (22–30)
*Primary admission diagnosis*	
**Medical illness (*n* [%])**	30 (77%)
Neurologic (*n*)	12
Respiratory (*n*)	6
Gastrointestinal (*n*)	5
Infection (*n*)	3
Shock (*n*)	2
Metabolic (*n*)	1
Vascular (*n*)	1
Oncologic (*n*)	0
**Surgery (*n* [%])**	6 (15%)
Neurosurgery (*n*)	3
General surgery (*n*)	2
Vascular surgery (*n*)	1
**Trauma (*n* [%])**	3 (8%)
Surgical (*n*)	2
Non‐surgical (*n*)	1
**Do not resuscitate order at time of death**	34 (87%)
**Median length of hospital admission** [Q1–Q3])	4 days (2–10)
**Median length of ICU admission** [Q1–Q3])	3 days (2–7)

Abbreviations: BMI, body mass index; ECG, electrocardiogram; ICU, intensive care unit.

Of the five participants with full code status, resuscitation was attempted for one participant prior to death. There were no instances of autoresuscitation.

**FIGURE 1:**
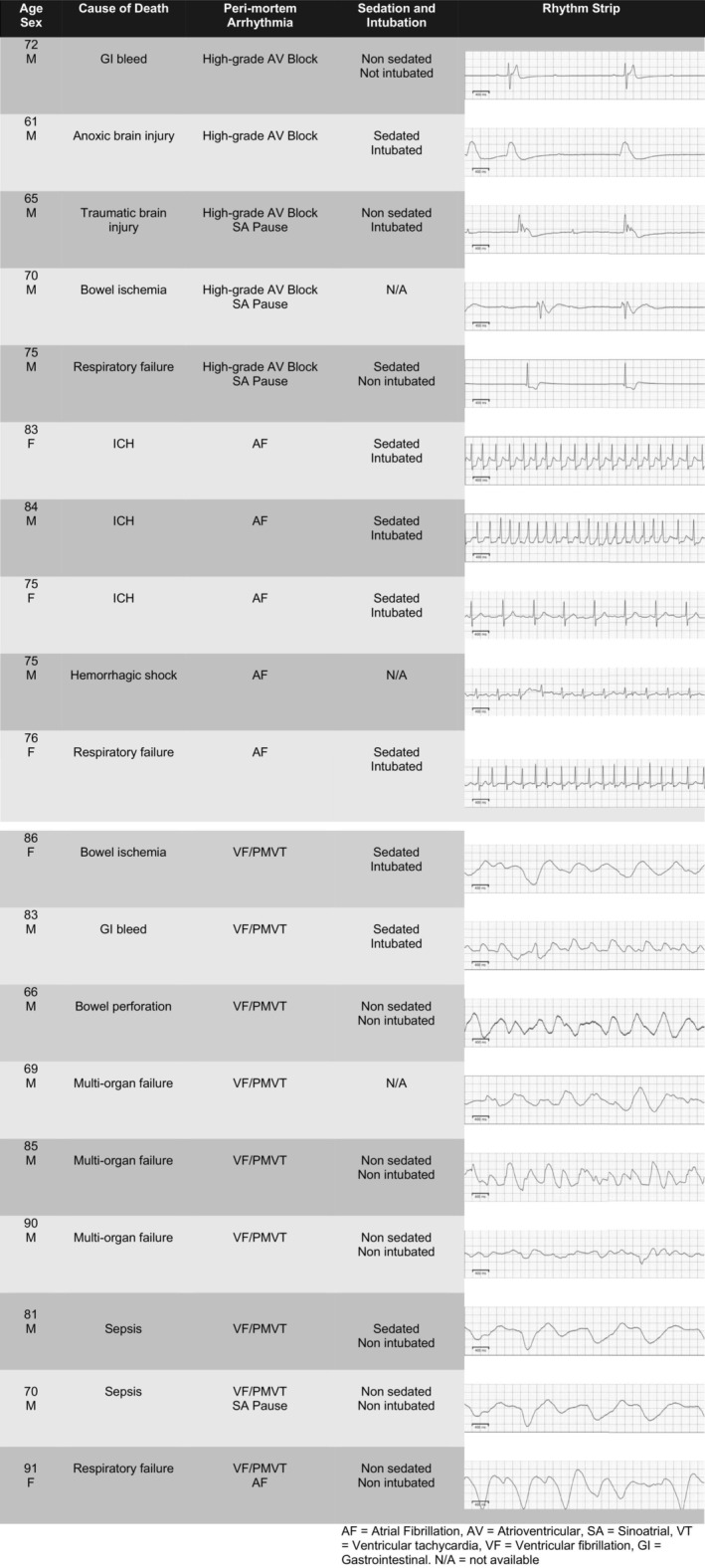
Peri‐mortem arrhythmias.

The incidence of sustained arrhythmia longer than 30 s in the last hour of life was 56%. Figure 1 provides details on the cause of death and sample rhythm strips. We observed nine instances of VF/PMVT (23%), seven sinoatrial pauses (18%), six instances of AF (15%) and five high‐grade AV blocks (13%; including 4 third‐degree AV blocks and 1 Mobitz type II second‐degree AV block). The patch captured a single arrhythmia in 17 participants, while 5 participants had two different arrhythmias. In participants who had more than one peri‐mortem arrhythmia, sinoatrial pause co‐occurred most often (80%).

AF was a terminal arrhythmia in all three participants with intracranial haemorrhage (ICH). VF co‐occurred with many different aetiologies of death, of which multi‐organ failure was the most common (33%).

## 4 DISCUSSION

This study identified that among patients who were admitted to the ICU and died in hospital, more than half (56%) of individuals developed at least one peri‐mortem arrhythmia.

To the best of our knowledge, this is the first systematic description of heart rhythms at the end of life in ICU patients. Our findings revealed that terminal arrhythmias occurred with a variety of admitting diagnoses and causes of death. The incidence of terminal rhythms has not been widely reported, although studies of cardiac implantable electronic devices (e.g. pacemakers, implantable cardioverter defibillators) have identified arrhythmias as the likely cause of death in 50%–70% of participants.^5^, ^6^, ^7^, ^8^ In one review of autoresuscitation cases, asystole was the most common rhythm at the termination of cardiopulmonary resuscitation (55%), followed by VF (18%) and pulseless electrical activity (14%).^9^

All but one of the participants in this study had a ‘do not resuscitate’ order at the time of death. The results of this study may thus be most useful for critically ill patients who are organ donation candidates. Current organ donation efforts are heralded by markers of clinical change, including agonal breathing and hemodynamic disturbances. Our study population comprised both awake and sedated participants, for whom death occurred within 1 h of these arrhythmias—this could potentially serve as a signal for organ donation teams to plan mobilization efforts. The appearance of arrhythmias may also serve as a marker of change in clinical status to update families who are anticipating the death of a loved one. In participants who are sedated or intubated, the development of novel arrhythmias could be a surrogate marker for respiratory or neurologic changes. Continuous cardiac monitoring and interpretation of electrophysiological changes may be useful for this purpose.

We included a diverse patient population, which increases the generalizability of our findings. This study is limited by a small sample size and the removal of some patches because of patient and family preference at the end of life. Future studies should be conducted with larger datasets to corroborate these findings and provide a basis for recommendations in clinical practice. Furthermore, it remains difficult to ascertain which rhythms are markers of imminent death rather than causative, so our findings are mainly descriptive.

## 5 CONCLUSIONS

Arrhythmias are common in dying critically ill patients across several mechanisms of death. There is a roughly even distribution between ventricular arrhythmias, AF, sinus node dysfunction and AV block.

## FUNDING INFORMATION

The AFOTS Incidence Study was funded in part by peer‐reviewed research grants from the Hamilton Health Sciences New Investigator Fund and the Canadian Stroke Prevention Intervention Network (C‐SPIN).

## CONFLICT OF INTEREST STATEMENT

Dr. McIntyre is supported by personnel awards from the Canadian Stroke Prevention Intervention Network and the Canadian Institutes for Health Research (CIHR). Dr. Belley‐Côté is supported by a personnel award from the McMaster University Department of Medicine and the Heart and Stroke Foundation of Canada. The remaining authors have disclosed that they do not have any potential conflicts of interest.

## ETHICS APPROVAL STATEMENT

The Hamilton Integrated Research Ethics Board approved this study.

## Data Availability

The data that support the findings of this study are available from the corresponding author upon reasonable request.
